# Treatment of Pulpectomized Teeth With Trypsin Prior to Transplantation of Mobilized Dental Pulp Stem Cells Enhances Pulp Regeneration in Aged Dogs

**DOI:** 10.3389/fbioe.2020.00983

**Published:** 2020-08-14

**Authors:** Koichiro Iohara, Mohammed Zayed, Yoshifumi Takei, Hideto Watanabe, Misako Nakashima

**Affiliations:** ^1^Department of Stem Cell Biology and Regenerative Medicine, National Center for Geriatrics and Gerontology, Research Institute, Obu, Japan; ^2^Department of Surgery, College of Veterinary Medicine, South Valley University, Qena, Egypt; ^3^Department of Medicinal Biochemistry, School of Pharmacy, Aichi Gakuin University, Nagoya, Japan; ^4^Institute for Molecular Science of Medicine, Aichi Medical University, Nagakute, Japan; ^5^Aeras Bio Inc., Air Water Group, Kobe, Japan

**Keywords:** aged teeth, allogeneic transplantation, mobilized dental pulp stem cells, pulp regeneration, trypsin

## Abstract

There is an age-dependent decline of pulp regeneration, due to the decline of migration, proliferation, and cell survival of resident stem cells. Trypsin is a proteolytic enzyme clinically used for tissue repair. Here, we investigated the effects of trypsin pretreatment of pulpectomized teeth prior to cell transplantation on pulp regeneration in aged dogs. The amount of regenerated pulp was significantly higher in trypsin-pretreated teeth compared to untreated teeth. Trypsin pretreatment increased the number of cells attached to the dentinal wall that differentiated into odontoblast-like cells. The trypsin receptor, PAR2, was higher *in vitro* expression in the periodontal ligament cells (PDLCs) from aged dogs compared to those from young. The direct effects of trypsin on aged PDLCs were increased expression of genes related to immunomodulation, cell survival, and extracellular matrix degradation. To examine the indirect effects on microenvironment, highly extracted proteins from aged cementum were identified by proteomic analyses. Western blotting demonstrated that significantly increased fibronectin was released by the trypsin treatment of aged cementum compared to young cementum. The aged cementum extract (CE) and dentin extract (DE) by trypsin treatment increased angiogenesis, neurite extension and migration activities as elicited by fibronectin. Furthermore, the DE significantly increased the mRNA expression of immunomodulatory factors and pulp markers in the aged DPSCs. These results demonstrated the effects of trypsin on the microenvironment in addition to the resident cells including PDLCs in the aged teeth. In conclusion, the potential utility of trypsin pretreatment to stimulate pulp regeneration in aged teeth and the underlying mechanisms were demonstrated.

## Introduction

The safety and efficacy of pulp regenerative therapy performed by the autologous transplantation of dental pulp stem cells (DPSCs) subsets, such as mobilized dental pulp stem cells (MDPSCs), was demonstrated in preclinical and clinical studies of young and middle-aged teeth (Nakashima and Iohara, 2017). MDPSCs were isolated based on their high migratory response to granulocyte-colony stimulating factor (G-CSF) (Murakami et al., 2013). They were determined to be similar between young and aged donors in terms of their trophic effects and stem cell properties *in vitro* and high regenerative potential in the ischemic hindlimb model and the ectopic tooth transplantation model (Horibe et al., 2014). However, there was an age-dependent decline in pulp regeneration after MDPSC transplantation in dog pulpectomized teeth. This decline was attributed in part to the reduced migration, proliferation, and cell survival of resident stem cells (Iohara et al., 2014). It is well known that with age, there is a gradual decline in the regenerative potential of most tissues due to age-dependent changes in the resident stem cells and the microenvironment or “niche” (Blau et al., 2015). With an increasingly aging population, a better understanding of the age-related changes in resident stem cell function and microenvironment is critical in developing and optimizing rejuvenation strategies to reverse the aging process for effective therapeutic treatment (Gibon et al., 2016). Aged teeth are characterized by a decrease in the regeneration of dental pulp (Iohara et al., 2014), stenosis and fibrosis of the periodontal ligament (Krieger et al., 2013), widening of the cementum, and constriction of the apical region (Jang et al., 2014), affecting resident stem cell function and the homeostasis of the tooth and associated tissue. Resident cells in the tissue surrounding the aged tooth have lower potential of migration, proliferation, and cell survival (Iohara et al., 2014). Many age-related diseases and aging itself are closely associated with low-level chronic inflammation (Jurk et al., 2014). It has also been demonstrated that resident cells are senescent under periapical chronic inflammation in the aged periapical tissue (Huttner et al., 2009).

Trypsin is a proteolytic enzyme that has been used clinically for over 40 years (Rajendran, 2018). Trypsin provides prompt and effective management of inflammatory symptoms and promotes the rapid recovery of acute tissue injuries to prevent progression to chronic injuries (Shah and Mital, 2018). An animal study on cartilage repair demonstrated the improved incorporation of the cartilage implant when treated with trypsin before being grafted onto a full-thickness articular cartilage defect (Chen et al., 2000). Trypsin also plays pivotal roles in cellular signal transduction mediated through the proteolytic activation of protease-activated receptors (PARs) (Zhao et al., 2014; Ramachandran et al., 2016). The activation of PAR2 reduces apoptosis (Bluff et al., 2008; Iablokov et al., 2014) and is involved in migration processes (Bluff et al., 2008). The modulatory role of PAR2 in inflammation was demonstrated in several models of inflammatory and autoimmune disease (Ramelli et al., 2010). The physiological responses to trypsin through the activation of PAR2 are associated with the inflammation process, and increased vascular permeability, blood vessel relaxation, hypotension, granulocyte infiltration, and release of cytokines have been demonstrated (Zhao et al., 2014; Rovai and Holzhausen, 2017). PAR2 is expressed in pulp cells subjected to caries lesions but is minimal in healthy pulp tissue. The activation of PAR2 by trypsin increased the *in vitro* expression of the proinflammatory mediator cyclo-oxygenase-2 (COX2), suggesting trypsin could be a potential therapeutic intervention target for pulpal inflammation (Lundy et al., 2010). Therefore, we hypothesized that trypsin pretreatment in the root canal might activate PAR2 in the resident cells of the periapical tissue to modulate periapical chronic inflammation in aged teeth.

It is well known that a reservoir of growth factors and cytokines are sequestered in the dentin matrix as signaling molecules (Smith et al., 2012; Schmalz et al., 2017). They are exposed or released in cases of disease or traumatic injury to the pulp and periodontal ligament (Smith, 2003; Silva et al., 2004). The potential involvement of these molecules in multiple functions, including migration, proliferation, differentiation, and anti-apoptosis (Smith et al., 2012), has provided new strategies for cell homing approaches to pulp regeneration with endogenous dentin matrix proteins (Widbiller et al., 2018). The ethylenediaminetetraacetic acid (EDTA) treatment of dentin promotes adhesion, migration and differentiation, angiogenesis, and neurogenesis (Qin et al., 2003). Thus, we hypothesized that an irrigation-based trypsin treatment in the root canal could release a variety of growth factors and cytokines from the dentin and cementum to support the migration, proliferation of resident stem/progenitor cells, immunomodulation/anti-inflammation, and anti-apoptosis, similar to EDTA treatment.

The aim of this study was to examine the effect of trypsin pretreatment prior to transplantation of MDPSCs on pulp regeneration in aged dog teeth. First, the efficacy and safety of the trypsin pretreatment were examined under optimal conditions. Next, the underlying mechanisms of trypsin was investigated. We hypothesized that there were direct and indirect effects. To investigate the direct effects of trypsin via PAR2, genes that were highly expressed after trypsin treatment were identified in the aged periodontal ligament cells (PDLCs), a representative of the resident cells in the tissue surrounding the teeth, by microarray and quantitative polymerase chain reaction analyses. The effect of trypsin pretreatment on the anti-apoptotic activity of the aged PDLCs was also examined. To investigate the indirect effects, a proteomic analysis was performed to identify the proteins highly extracted from aged cementum. The aged cementum extract (CE) and dentin extract (DE) treated with trypsin were further examined with regard to enhanced angiogenesis, neurite extension and cell migration. Aged DE were also examined in the immunomodulatory and pulp regenerative effects. Thus, the potential utility and underlying mechanism of a trypsin pretreatment for pulp regeneration in aged teeth was demonstrated.

## Materials and Methods

All animal procedures were approved by the Animal Care and Use Committee of the National Center for Geriatrics and Gerontology, Research Institute and the Aichi Medical University (permission # 2016-5, 2017-25).

### Cell Culture

MDPSCs were isolated by G-CSF-induced stem cell mobilization method as described previously (Murakami et al., 2013) from the upper teeth of middle-aged dogs (*n* = 3) for the *in vivo* study. DPSCs and PDLCs were isolated from the extracted upper teeth of aged (5–6 year-old) and young (8–10-month-old) dogs (*n* = 3, each), cultured in DMEM supplemented with 10% fetal bovine serum (FBS, GE Healthcare UK Ltd., Little Chalfont, United Kingdom) and cryopreserved at the 4th–7th passage of culture for the *in vitro* studies. For experiments, DPSCs and PDLCs were seeded in 60 mm dishes (Falcon, Corning, United States) at a density of 2 × 10^5^ cells/dish. When the cells reached confluence, different treatments were applied.

### Trypsin Pretreatment for Pulp Regeneration of MDPSC Transplantation

Cell transplantation for pulp regeneration was performed in pulpectomized teeth (upper 1st and 2nd incisors and lower 2nd and 3rd incisors from right and left sides were used) as described previously (Iohara et al., 2013) with a slight modification. A total of 25 teeth from 12 aged female dogs (Kitayama Labes, Ina, Japan) at 5–6-year-old were used for the allogeneic transplantation of MDPSCs with G-CSF (Neutrogin, Chugai Pharmaceutical, Tokyo, Japan) and an atelocollagen scaffold (Koken, Tokyo, Japan). Prior to cell transplantation, the root canal was irrigated with 6% NaOCl and 3% H2O2 and Saline, and further with 3% EDTA solution (SmearClean, Nippon Shika Yakuhin Co., Ltd., Shimonoseki, Japan) for 2 min. The trypsin pretreatment (Francetin-T-powder, Mochida Pharmaceutical, Tokyo Japan) was performed in the aged teeth: group I, no treatment (*n* = 4); group II, nanobubble treatment (*n* = 4); group III, 0.05% trypsin for 10 min (*n* = 4); group IV, 0.5% trypsin for 10 min (*n* = 4); group V, 0.05% trypsin for 30 min (*n* = 4); group VI, 0.05% trypsin for 10 min with nanobubbles (*n* = 5). Nanobubbles were produced in the FOAMEST 8^®^ Nanobubble Generator by a nanoscale porous polymer film (Nac Corp. Seki, Japan). Nanobubbles contain pressurized air and are approximately 100–200 nm in diameter and have a negatively charged surface and Zeta potential of −18 ∼−22 mV. The enhanced delivery of medicaments by nanobubbles into dentinal tubules via irrigation has been demonstrated (Iohara and Nakashima, 2019). We used nanobubbles to enhance the delivery of trypsin into the dentinal tubules. As a control, a total of 4 teeth from 2 young dogs at 8–10-month-old were used for group VI (0.05% trypsin for 10 min with nanobubbles). All of the teeth were extracted on day 14, and the other teeth for group VI in the aged dogs were further extracted at 36 weeks.

### Histological and Immunochemical Analyses

Histological examination of the regenerated tissue was performed in the paraffin sections (5μm in thickness) of the teeth. The regenerated tissue was outlined in on-screen image of the histological preparations by a binocular microscope (Leica M 205 FA, Leica Microsystems, Wetzlar, Germany) and its relative amount to the root canals was determined by using Leica Application Suite software (Leica, version 3.4.1). For neovascularization and innervations analyses, Fluorescein Griffonia (Bandeiraea) Simplicifolia Lectin 1/fluorescein-galanthus nivalis (snowdrop) anti-PGP9.5 (Ultra Clone) (1: 10,000) was used, respectively in 5-μm-thick paraffin sections. The ratios of newly formed capillary area and neurite extension area to the regenerated pulp area and the periapical area were measured, respectively by Dynamic cell count BZ-HIC (KEYENCE, Osaka, Japan). Masson trichrome staining (Muto pure chemicals Co., Ltd. Tokyo, Japan) was also performed in each 4 sections at 2 weeks (*n* = 4) for quantitative analysis of matrix formation.

### Safety Assessment

For the toxicology assessment, the clinical signs, food consumption, and weights were observed weekly. The examinations of urine and blood chemistry were performed before transplantation and at 1, 4, and 12 weeks after transplantation. Furthermore, the periapical tissue of the trypsin pretreated teeth was histopathologically examined in paraffin sections with H.E. staining. X-ray analysis (Morita, Osaka, Japan) was also performed at 9 months to examine the periapical region.

### Microarray Analysis

To examine the direct effect of trypsin, PDLCs from aged dog teeth were treated with 0.05% trypsin or PBS as a negative control for 3 min. Furthermore, mRNA was extracted, and highly expressed genes in trypsin-treated PDLCs compared to control were identified by a microarray analysis as described previously (Ishizaka et al., 2012) with a slight modification (additional details are given in Supplementary Material).

### The Direct Effects of Trypsin Treatment in Aged PDLCs

The expression of the trypsin receptor, PAR2 protein was examined in aged PDLCs compared to young PDLCs by Western blot analysis. To examine the direct effect of trypsin, PDLCs (*n* = 3, each) from aged and young dog teeth were treated at confluency with 0.05% trypsin or PBS as a negative control for 3 min, and mRNA was extracted from each cell. The canine primers of highly expressed genes in aged PDLCs treated with trypsin identified by the microarray analysis were constructed and real-time PCR were performed to examine the mRNA expression of immunomodulatory, tissue repair-related, and anti-apoptotic factors (Table 1). In addition, to examine the direct effect of trypsin on cell survival, Bcl-2 and Bax expression, extracted proteins from aged and young PDLCs treated with trypsin were compared with non-treated control by Western blot analyses (details of real-time PCR, see section “Real-Time Polymerase Chain Reaction” and for Western blot analyses, see section “Western Blot Analyses”).

**TABLE 1:** Canine primer sequences used in real-time polymerase chain reaction analysis.

Primer name		Primer sequence	Size
IDO	Forward	GGAAAGGCAACTCCAAACTG	124 bp
	Reverse	CCCAGCAGAATGTCAAAGC	
TGF-β1	Forward	CTGGAGTCGTGAGGCAGTG	96 bp
	Reverse	GCAGTGTGTTATCTTTGCTGTCA	
PTGE	Forward	GCCGCTGTGACTGTACC	190 bp
	Reverse	TGGTCCAATCAGCCACTTC	
TRH-DE	Forward	CCAGCAGGCATCAACACTTA	136 bp
	Reverse	CCTGTCATCGCTGCAAGTTA	
Syndecan	Forward	TCATGCAGGACAGCTTCAAC	186 bp
	Reverse	AGGGCTGGAATCTAGGGAAA	
Tenascin	Forward	TGGCTGTCTTGGACACAGAG	181 bp
	Reverse	GACTCCAGAGTTGGGGTCTG	
NCF1	Forward	TACCGTGCCATTGCTGACTT	125 bp
	Reverse	CGCTTTGTCTTCGTTTGGCA	
TOM1L1	Forward	CAGTGAACCTTCTGCCCCAT	135 bp
	Reverse	ACAGGACCTGGAGAGCTGAA	
CB2R	Forward	GTCTTCGCCTTCTGCTCCTT	126 bp
	Reverse	CAGATGCCTTCTCCAGTGGG	
TSP1	Forward	GTTCTGGCTGCCCAAACTTG	148 bp
	Reverse	CCACGTCTCCTTGCTTGTCT	
IL-18	Forward	GCGGAAAGTGATGAAGGCCT	148 bp
	Reverse	CTGTACAGTCAGAATCGGGCA	
AMPD2	Forward	AAAGTCCTTCTGCTACCGCC	137 bp
	Reverse	TGTCCACCTTCCGGATGTTG	
VWA3B	Forward	ACTTGTGGCATCCCGGTAG	148 bp
	Reverse	CCGCTGCTTGTAACTCTCCA	
β-actin	Forward	AAGTACCCCATTGAGCACGG	257 bp
	Reverse	ATCACGATGCCAGTGGTGCG	

*IDO, indoleamine 2,3-dioxygenase; TGFβ1, transforming growth factor-beta1; PTGE, prostaglandin E synthase; TRH-DE, Thyrotropin Releasing Hormone Degrading Enzyme; NCF1, Neutrophil Cytosolic Factor 1; TOM1L1, target of Myb 1; CB2R, Cannabinoid Receptor 2; TSP1, Thrombospondin 1; IL-18, Interleukin-18; AMPD, Adenosine Monophosphate Deaminase; VWA3B, Von Willebrand Factor A Domain Containing 3.*

### Proteomic Analysis

The teeth were extracted from aged and young dogs and transported in Hank’s balanced salt solution. After removal of the periodontal ligament, cementum was treated with 0.05% trypsin for 10 min and the proteins were extracted using the specific proteomic solution tris-based. The protein concentration was measured by a Bradford assay. The purified proteins were subjected to SDS-PAGE followed by Coomassie brilliant blue (CBB) staining, and the bands were excised from the gel for mass spectrometry (MS) analysis as described previously with a slight modification (Murakami et al., 2012) (additional details are given in Supplementary Material).

### Preparation of the CE and DE

Upper dog teeth were extracted from aged (5–6 years) and young (8–10 months) dogs. To obtain the CE, the cementum was treated with Smearclean (3%EDTA, Nippon Shika Yakuhin, Shimonoseki, Japan) for 2 min, and further extracted in 0.05% trypsin for 10 min. To obtain the DE, the dentin was cut into small pieces after complete removal of the enamel, pulp, cementum and periodontal ligament from the teeth, treated with Smearclean, and extracted for 30 min in 0.05% trypsin, 10% EDTA (17%EDTA liquid, Pentron Japan, Tokyo, Japan), 0.05% chymotrypsin (Chymotrypsin sequence grade, Sigma Aldrich, St. Louis, MO, United States), or PBS as a negative control, respectively. The CE and DE were concentrated via centrifugation in an Amicon Ultra 3K device (Millipore, Darmstadt, Germany). The protein concentration was measured by using a Bradford Ultra^TM^ kit (Expedeon, Cambridge, United Kingdom). Western blot analyses of fibronectin were performed in the CE and DE from aged and young teeth treated with trypsin, and further in aged and young DE treated with trypsin, chymotrypsin, 10% EDTA, and PBS control. Protein levels were normalized to β-actin for quantification (details of Western blot analyses, see section “Western Blot Analyses”).

### Real-Time Polymerase Chain Reaction

Total RNA was extracted with TRIzol (Thermo Fisher Scientific, Waltham, MA). First strand cDNA was generated using 1μg of total RNA by reverse transcription using the ReverTra Ace-α (Toyobo, Tokyo, Japan) according to the manufacturer’s protocol. Reverse-transcribed products were amplified by the SYBR method using 7500 real-time PCR system (Applied Biosystems, Foster City, CA) according to the manufacturer’s instruction. To examine mRNA expression of genes related to senescence, anti-apoptosis, anti-inflammation/immunomodulation, and extracellular matrix degradation, a real-time PCR amplifications of dog primers were performed (Table 1). Threshold cycle number (CT) was automatically determined by ABI 7500 software and mRNA expression was normalized with β-actin.

### Western Blot Analyses

The expression of PAR2, Bcl-2, Bax and Fibronectin were analyzed by Western blotting. Proteins were concentrated using an ultrafiltration unit with a 3 kDa molecular weight cut-off (Amicon Ultra-15 Centrifugal Filter Unit with Ultracel-3 membrane) (Millipore, Billerica, MA) and stored with proteinase inhibitors (Halt^TM^ proteinase inhibitor cocktail EDTA-free, Thermo Scientific, Rockford, IL) at −30° until use. Protein concentration was determined by Bradford Ultra^TM^ (Expedeon, Cambridge, United Kingdom). After denaturation at 95°, protein samples were electrophoresed on a 12% polyacylamide gel and the separated proteins were transferred onto a PVDF membrane. For each analysis, PAR2 (1:250, Santa Cruz Biotechnology, Dallas, TX), Bcl-2 (1:500, BD Biosciences, San Jose, CA), Bax (1:500, BD Biosciences), Fibronectin (1:1000, abcam, Cambridge, MA, United States) and β-actin (1:1000, Cell signaling technology, Danvers, MA, United States) were incubated overnight at 4°C to detect the target protein. Antigen detection was performed using anti-rabbit secondary horseradish peroxidase-conjugated antibody followed by exposure to Luminata Forte Western HRP Substrate (Merck, Darmstadt, Germany). The intensity of the immunoactivity obtained for each protein was quantified by densitometry using ImageJ software (version 1.52, imagej.nih.gov). Protein levels were normalized to β-actin for quantification.

### Effect of CE or DE on Angiogenic, Neurogenic, and Migration Activity

The angiogenic, neurite extension and migratory effects of aged CE and DE treated with 0.05% trypsin were evaluated and compared with the effects of fibronectin only and G-CSF/BDNF as a positive control.

For angiogenesis, human umbilical vein endothelial cells (HUVEC, 6 × 10^4^/well) (Lonza, Basel, Switzerland) were seeded on 48-well plates. The cells were seeded on Matrigel (BD Biosciences) in DMEM containing 2% FBS, 5 μg/ml heparin (Lonza), 5 μg/ml ascorbic acid (Lonza), 5 μg/ml hydrocortisone (Lonza) supplemented with DE or CE or fibronectin (5 μg/ml). G-CSF (100 ng/ml) was used as a positive control. The mean length of networks of cords and tube-like structures were measured 5 h after cultivation under an inverted microscope (Leica, 6000B-4, Leica) using ImageJ software (version 1.52, imagej.nih.gov).

For the quantification of neurite outgrowth, human neuroblastoma cell line TGW were plated in 24-well plates at a density of 1 × 10^5^ cells/ml. The cells were starved overnight and then stimulated with DE or CE, fibronectin (5 μg/ml) for 24 h. The mean neurite length was measured under the inverted microscope using ImageJ software. The same experiment was performed with 50 ng/ml brain-derived neurotrophic factor (BDNF) (Peproteck, London, United Kingdom).

Chemotactic effect of DE and CE treated with 0.05% trypsin and fibronectin were evaluated in aged PDLCs by a modified Boyden chamber assay. PDLCs were seeded at the cell concentration of 1 × 10^5^ in 100 μl of DMEM on the top of an insert membrane with 8 μm pore size in 24-well plates (Corning-Transwell-polycarbonate membrane cell culture inserts, Sigma-Aldrich). The lower compartment medium contains 2% FBS and supplemented with DE or CE or fibronectin 5 μg/ml. DMEM with 100 ng/ml of G-CSF (positive control) and 2% FBS only were used as a negative control. After 24 h, cells attached on the upper surface of the insert membrane were removed by cotton swabs. The attached cells on the lower surface of the membrane were fixed and stained with Giemsa. After washing, the stained cells were counted under an inverted bright-field microscope at × 100 magnification. Each experiment was performed in triplicate.

Effect of DE treated with trypsin, EDTA and PBS control on the relative gene expression of immunomodulatory factors and pulp tissue markers was examined by real-time PCR analyses both in young and aged DPSCs and young and aged PDLCs (details, see section “Real-Time Polymerase Chain Reaction”).

### Attachment of Pulp Cells Onto Dentin

DPSCs were cultured on dentin slices (100 μm) treated with 0.05% trypsin or 10% EDTA for 30 min. After 24 h, the slices were fixed and stained with 0.5% (w/v) crystal violet in methanol for 10 min. The number of cells attached to the dentin was counted by stereo microscopy (Leica M205 FA).

To further examine the odontoblastic differentiation of DPSCs after attachment to the dentin, pellet culture was performed together with the dentin which were pretreated with 0.05% trypsin, 10% EDTA or PBS for 10 min, and examined histologically in paraffin sections with H.E. staining at 4 weeks.

### Statistical Analyses

All of the results were expressed as the means ± standard deviation. The data were analyzed statistically using a *t*-test or one-way analysis of variance (ANOVA) with Tukey’s comparison test as a posttest using SPSS 21.0 (IBM, Armonk, NY, United States).

## Results

### Trypsin Pretreatment Stimulates Pulp Regeneration in Aged Dog Teeth

Our recent histological study on the microenvironment of aged teeth compared to young teeth has demonstrated that the stenosis and fibrosis of the periodontal ligament and hypercementosis, constriction of the apical region, and mineralization of pulp tissue (Zayed et al., 2020). In the microenvironment of the aged teeth, the fibrous tissue in the apical region may possibly prevent resident cells from migration from the surrounding tissue into the root canal and regeneration of pulp tissue.

**FIGURE 1:**
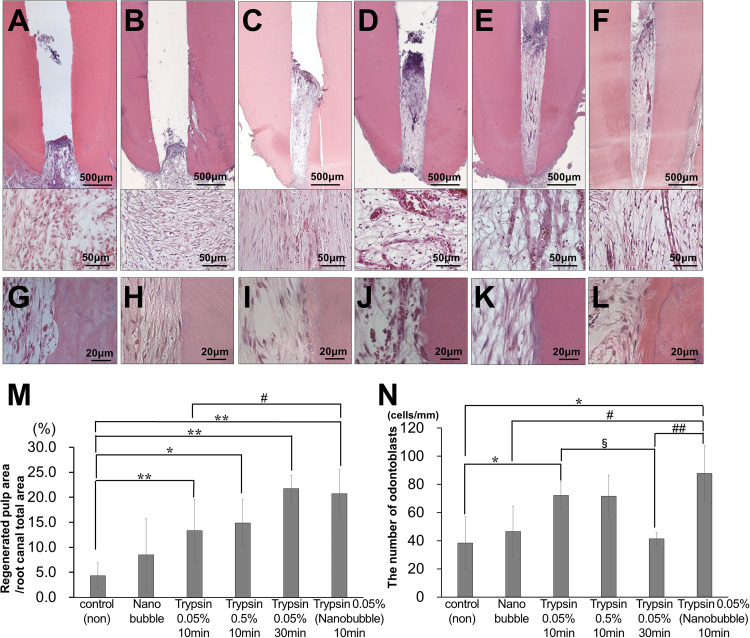
Optimal conditions of trypsin pretreatment of pulpectomized teeth prior to cell transplantation in aged dogs. **(A–F)** Pulp regeneration. **(G–L)** Attached cells on the dentinal wall. **(A,G)** Control. **(B,H)** Nanobubbles. **(C,I)** 0.05% trypsin for 10 min. **(D,J)** 0.5% trypsin for 10 min. **(E,K)** 0.05% trypsin for 30 min. **(F,L)** 0.05% trypsin with nanobubbles for 10 min. **(M)** Ratio of newly regenerated area to root canal area. **(N)** The number of odontoblast-like attached cells on dentin per mm. ^∗^*p* < 0.05, ^∗∗^*p* < 0.01 versus control. ^#^*p* < 0.05, ^##^*p* < 0.01 versus 0.05% trypsin with nanobubbles for 10 min. ^§^*p* < 0.05 versus 0.05% trypsin for 30 min. Data are expressed as the means ± standard deviation (*n* = 4).

Thus, we examined the effect of trypsin pretreatment prior to MDPSC transplantation on promoting pulp regeneration in aged dog teeth (Figures 1A–N). First, we optimized the conditions of trypsin pretreatment. We compared the amount of regenerated pulp tissue in the pulpectomized teeth at 14 days after transplantation. Compared to lack of pretreatment by trypsin, the amount of pulp-like regenerated tissues were three-times higher with 0.05 and 0.5% trypsin pretreatment for 10 min, respectively (Figures 1A,C,D,M). The volume of regenerated pulp tissue by 0.05% trypsin was increased by 30 min treatment compared to 10 min (Figures 1C,E,M). Next, we compared 0.05% trypsin pretreatment with and without nanobubbles for 10 min. Nanobubbles have previously demonstrated to enhance the permeation of medication into the dentin (Iohara and Nakashima, 2019). The volume of regenerated pulp tissue was approximately twice higher with the pretreatment consisting of nanobubbles (Figures 1C,F,M), indicating that nanobubbles increased the amount of regenerated pulp tissue. However, there was no significant difference between with and without nanobubbles for 0.05% trypsin pretreatment for 10 min in the number of odontoblast-like cells and osteodentinoblast-like cells attached to the wall of newly formed dentin (Figures 1I,L,N). On the other hand, in young dogs, there was no significant difference in the volume of regenerated pulp tissue between the 0.05% trypsin pretreatment with nanobubbles for 10 min and no pretreatment (Supplementary Figure 1).

**FIGURE 2:**
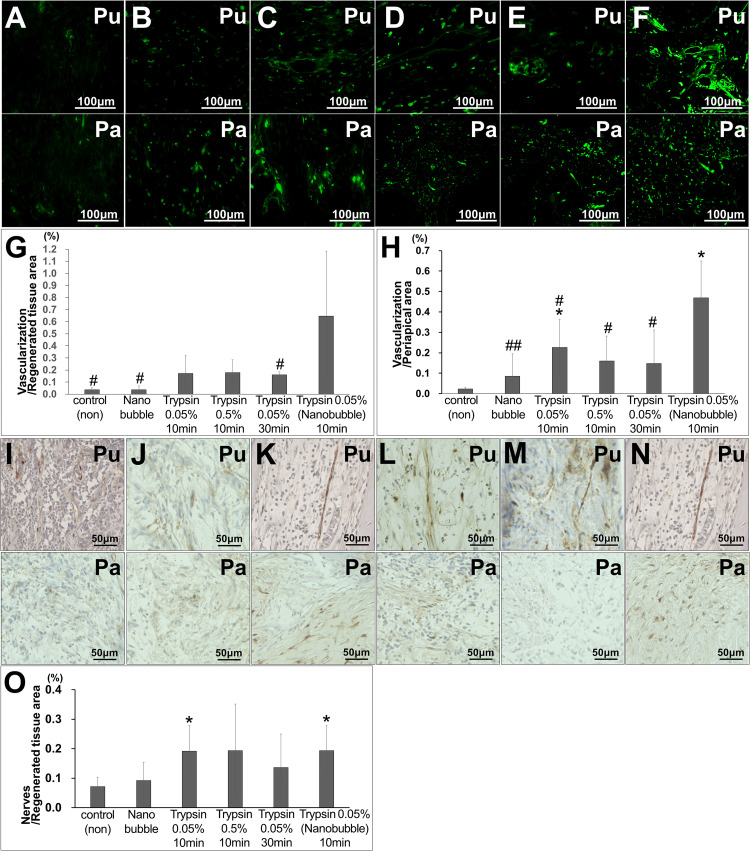
Angiogenesis and neuronal extension in the regenerated pulp tissue and periapical region. **(A–F)** BS-1 lectin staining. **(G,H)** Ratio of positively stained area by BS-1 lectin. *p < 0.05, versus control (non). ^#^*p* < 0.05, ^##^*p* < 0.01, versus trypsin 0.05% with nanobubble. Data are mean ± standard deviation (*n* = 4). **(G)** Regenerated pulp tissue area. **(H)** Periapical region area. **(I–N)** PGP9.5 staining. **(O)** Ratio of positively stained area by PGP 9.5 in the regenerated tissue pulp area. Data are expressed as the means ± standard deviation (*n* = 4). *p < 0.05, versus control (non). **(A,I)** Control. **(B,J)** Nanobubble. **(C,K)** 0.05% trypsin for 10 min. **(D,L)** 0.5% trypsin **(E,M)** 0.05% trypsin for 30 min. **(F,N)** 0.05% trypsin with nanobubbles for 10 min. Pu, Regenerated pulp; Pa, periapical region.

In the regenerated pulp tissue and periapical tissue, the trypsin pretreatment induced higher vascularization compared with no pretreatment (Figures 2A–G). The pretreatment with 0.05% trypsin with nanobubbles for 10 min induced the highest density of blood vessels in the regenerated pulp tissue (Figure 2G). The vascularization was 4 times and 3 times higher in the regenerated pulp tissue and in the periapical tissue, respectively, for the 0.05% trypsin pretreatment with nanobubbles for 10 min compared with the 0.05% trypsin pretreatment for 30 min. The 0.05% pretreatment with nanobubbles for 10 min induced the highest vascularization among all of the pretreatments also in the periapical tissue (Figure 2H). In the regenerated pulp tissue, the pretreatment with 0.05% trypsin with or without nanobubbles for 10 min induced a higher neurite extension compared with no pretreatment (Figures 2I–O). Thus, the 0.05% trypsin pretreatment with nanobubbles for 10 min was considered to be the optimum for pulp regeneration.

**FIGURE 3:**
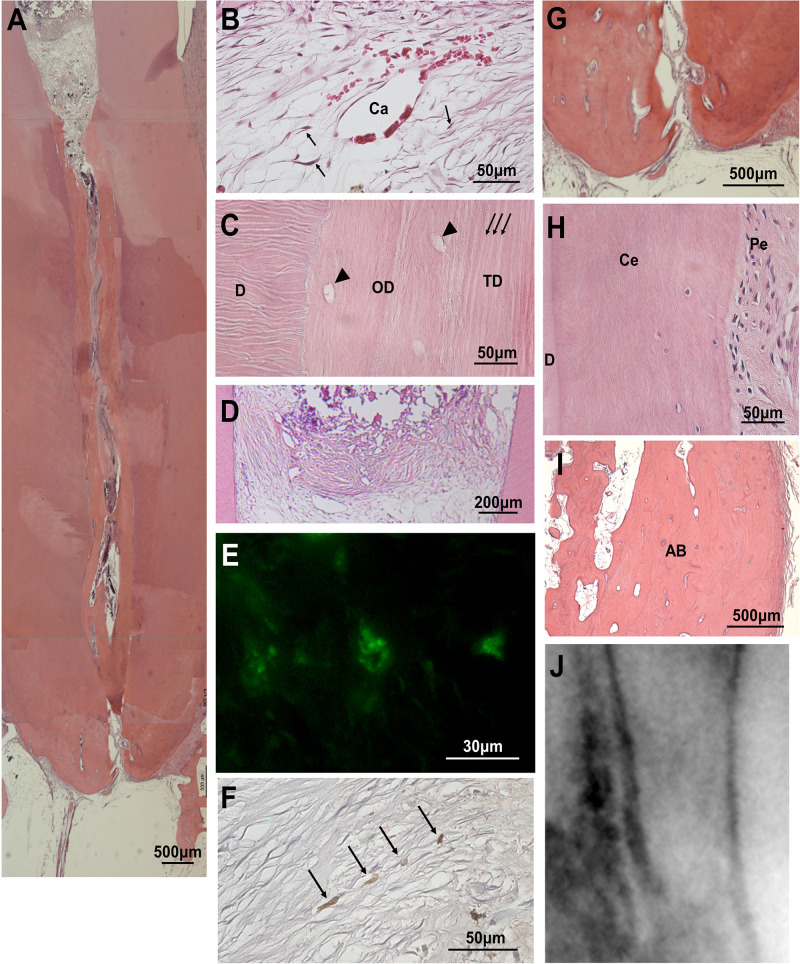
Regenerated pulp tissue at 36 weeks after cell transplantation. **(A)** A large amount of dentin-like mineralized tissue is formed along the dentinal wall. **(B)** Regenerated pulp tissue. Spindle-shaped pulp-like cells (arrows) and a capillary (Ca). **(C)** Higher magnification of osteodentin-like mineralized tissue (OD) and tubular dentin-like mineralized tissue (TD) formation. Osteodentinocytes in the lacunae (arrow heads). Dentinal tubules (arrows). Original dentin (D). **(D)** Osteodentin-like matrix formation on the regenerated pulp in the crown part. **(E)** BS1-lectin staining for angiogenesis. **(F)** PGP9.5 staining for neurite extension (arrows). **(G)** Periapical tissue. **(H)** Periodontal ligament (Pe) in the periapical region. Cementum (Ce). Original dentin (D). **(I)** Alveolar bone (AB). **(J)** X-ray analysis in the periapical region.

The pulp-like tissue was further regenerated to the cementum-enamel junction below the cement filling at 36 weeks after MDPSC transplantation with pretreatment with 0.05% trypsin with nanobubbles for 10 min (Figures 3A,B), indicating complete pulp regeneration. Osteodentin and/or tubular dentin-like mineralized tissue formation was observed along the dentinal wall (Figure 3C). In the coronal part of the root canal, a small amount of osteodentin-like tissue was formed on the surface of the regenerated pulp tissue (Figure 3D). Newly formed vessels stained by BS-1 lectin were observed in the regenerated tissue (Figure 3E). Nerve fibers positively stained by the PGP9.5 antibody were extended into the newly regenerated tissues (Figure 3F). In the apical region of the root canal, the physiological apical foramen was greatly decreased in diameter by the additional formation of dentin and cementum (Figure 3G).

### Safety of Trypsin Pretreatment Prior to Cell Transplantation

The safety of MDPSC transplantation in the pulpectomized teeth has already been demonstrated in preclinical and clinical studies (Iohara et al., 2013; Nakashima et al., 2017). Thus, the safety of trypsin pretreatment prior to MDPSC transplantation was also examined by a toxicology assessment. There were no adverse effects on appearance, clinical signs, food consumption or body weight for 12 weeks after transplantation that was followed by a 0.05% trypsin pretreatment with nanobubbles for 10 min. Serum and urine chemistry parameters showed values within normal ranges at 12 weeks, demonstrating no immunoreaction to the trypsin pretreatment prior to cell transplantation (Table 2). No infiltration of inflammatory cells was observed in the periapical tissue, such as in the periodontal ligament or alveolar bone at 36 weeks (Figures 3H,I). The X-ray photographic analysis at 36 weeks showed no significant changes in the periapical areas related to the cell therapy with trypsin pretreatment (Figure 3J).

**TABLE 2:** Safety evaluation by hematology and blood chemistry at 1, 4, and 12 weeks after trypsin pretreatment and MDPSC transplantation.

	Before pretreatment	1 week	4 weeks	12 weeks	Normal
**Blood test**					
TP	5.7–6.4	6.3–7.1	5.5–6.3	6.8–7.3	6–8
Albumin	2.5–2.7	2.7–3.0	2.4–2.7	3.0–3.1	2.5–3.6
A/G	0.73–0.84	0.73–0.81	0.70–0.82	0.73–0.79	0.45–1.19
GPT(ALT)	20–29	23–48	27–36	27–34	18–54
GOT(AST)	39–77	86–119	40–57	48–63	20–70
ALP	181–254	164–253	177–211	131–168	104–239
T-Bil	0	0	0	0	0.1–0.5
Cre	0.59–0.76	0.53–0.75	0.56–0.87	0.53–0.74	0.6–0.9
UN/Cr	11.2–15.8	9.1–16.0	13.8–21.3	24.7–29.8	9.2–29.2
Na	144–147	143–151	144–145	139–146	133–146
Cl	108–110	107–112	112–113	104–108	105–115
K	4.5–5.0	4.4–5.1	4.3–5.2	4.6–4.8	3.9–5.1
CRP	< 0.05	< 0.05	<0.05	<0.05	<1.0
**Urinalysis**					
Specific gravity	1.025–1.050	1.013–1.034	1.004–1.010	1.009–1.031	1.008–1.05
PH	9.0–9.0	9.0–9.0	5.0–8.5	9.0–9.0	4.8–7.5
Urobilinogen	+−	+−	+−	+−	+−
Bilirubin	−	−	−	−	−
Ketone body	−	−	−	−	−
Color	Yellow	Yellow	Yellow	Yellow	Yellow

### Direct Effects of Trypsin Treatment on Aged PDLCs *in vitro*

**FIGURE 4:**
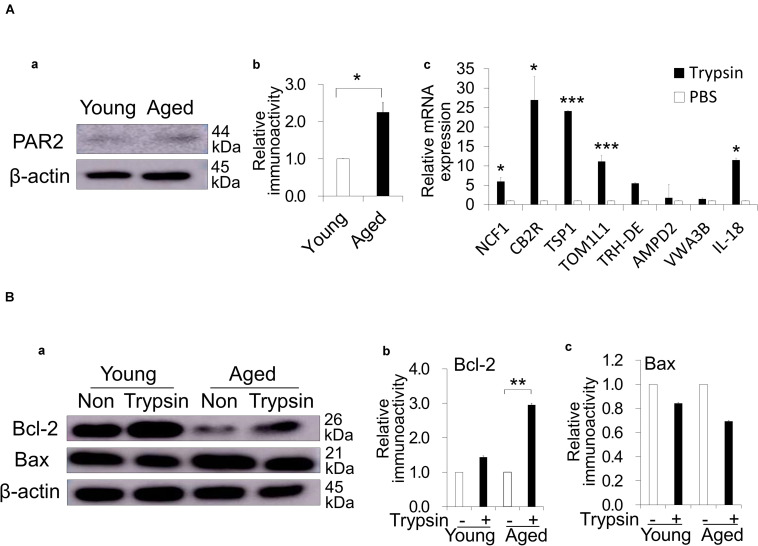
Direct effect of trypsin treatment in dog periodontal ligament cells (PDLCs). **(Aa)** Protease activated receptor 2 (PAR2) expression in the aged PDLCs compared with the young PDLCs by Western blot analysis. **(b)** The quantitative analyses of PAR2 immunoblot. *p < 0.05. **(c)** Real-time reverse transcription-polymerase chain reaction analysis of highly expressed genes identified by the microarray analysis in the aged PDLCs treated with 0.05% trypsin compared to PBS control. *p < 0.05, ***p < 0.001 versus PBS control. **(Ba)** Bcl-2 and Bax expression in the young and aged PDLCs treated with trypsin compared with PBS control by Western blot analyses. **(b)** The quantitative analyses of Bcl-2 and **(c)** Bax immunoblot. **p < 0.01. All data are expressed as the means ± standard deviation (*n* = 3).

To demonstrate the direct effects of the trypsin treatment, the expression of a main trypsin receptor, PAR2, was examined by immunoblotting analysis in PDLCs—the resident cells in periapical tissue. As expected, the aged PDLCs showed higher expression of PAR2 compared with young PDLCs (Figures 4Aa,b). The highly expressed genes in the aged PDLCs after direct trypsin treatment *in vitro* were examined by a microarray analysis. The results demonstrated that the genes related to senescence (*TSP1*), anti-apoptosis (*VWA3B*), anti-inflammation/immunomodulation (*NCF1*, *IL18*, and *CNR2/CB2R*), and extracellular matrix degradation (*TOM1L1*) are highly expressed (Supplementary Table 1). Quantitative PCR further confirmed that the aged PDLCs with trypsin treatment have significantly higher gene expression levels of *NCF1*, *IL18*, *CNR2/CB2R*, *TSP1*, *TOM1L1* compared with the PBS control (Figure 4Ac). To show the direct effect of trypsin pretreatment on anti-apoptosis, a Western blot analysis demonstrated that the cell survival protein Bcl-2 was increased in the aged PDLCs by trypsin treatment (Figures 4Ba,b), indicating the inhibition of apoptosis. There was no difference in the expression of Bax between the aged and young PDLCs (Figures 4Ba,c).

### Indirect Effects of CE and DE Treated With Trypsin

**FIGURE 5:**
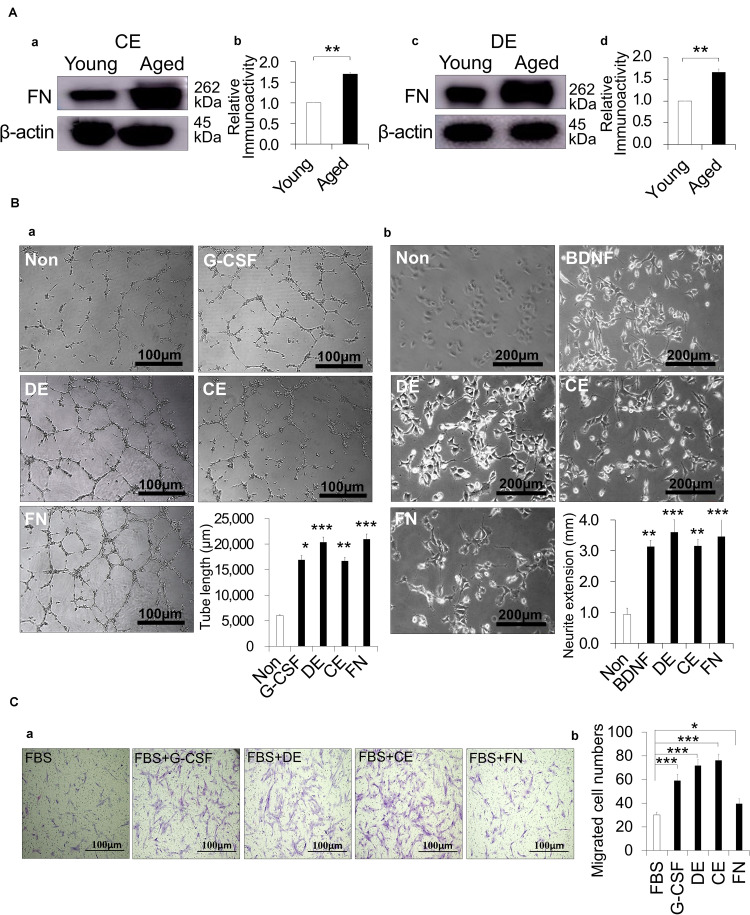
Indirect effect of cementum extract (CE) and dentin extract (DE) treated with 0.05% trypsin on angiogenesis, neurite extension and migration activities. **(A)** Release of fibronectin (FN) from dog CE and DE with 0.05% trypsin for 10 min and 30 min, respectively. Western blot analysis showing FN expression in **(a,b)** young and aged CE, and **(c,d)** young and aged DE. **(b,d)** The quantitative analyses of FN. **p < 0.01. **(Ba)** Angiogenesis activity of DE, CE, FN, and granulocyte-colony stimulating factor (G-CSF) on HUVEC, showing network formation after 5 h. Statistical analysis of the length of the tubes. **(b)** Neurite extension activity of DE, CE, FN, and BDNF on TGW cells. Statistical analysis of neurite length for the different conditions. **(Ca)** Migration activity of DE, CE, FN, and G-CSF on aged PDLCs. **(b)** Statistical analysis of migration activity after 24 h. *p < 0.05, **p < 0.01, and ***p < 0.001. All data are expressed as the means ± standard deviation (*n* = 3).

The indirect effect of trypsin treatment was examined by a proteomic analysis to identify the proteins that were highly extracted from cementum of aged dogs by the trypsin treatment. The increase of many cytoskeletal components such as Filamin A, Plectin, Talin, Alpha-actinin, microtubule-associated protein 1B and Vinculin; ECM proteins including fibronectin, collagen alpha-1 and 3 (XII, VI); and signaling molecule, Clathrin heavy chain 1 was demonstrated (Supplementary Table 2). A Western blot analysis demonstrated that the trypsin treatment of aged cementum significantly released more fibronectin compared with young cementum (Figures 5Aa,b). Fibronectin was also highly released from aged dentin (Figures 5Ac,d).

Next, the indirect effects of the aged CE and aged DE on angiogenesis, neurite extension and migration were examined in comparison with fibronectin. For angiogenic induction, there were more extensive networks of cords and tube-like structures in HUVEC treated with both CE and DE compared with the untreated control, similar to the result observed after treatment with fibronectin. The tube formation length was significantly higher for CE and DE as well as for fibronectin compared with the untreated control (Figure 5Ba). For neurogenic induction, the treatment of TGW cells with CE, DE and fibronectin enhanced neurite extension. The statistical analysis demonstrated that the neurite outgrowth was significantly higher in the TGW cells treated with CE, DE and fibronectin compared to the untreated cells (Figure 5Bb). There were no significant differences in the induced activities of angiogenesis and neurite extension among CE, DE and fibronectin. Promoting the migration of endogenous stem cells of the surrounding tissue, such as the periodontal ligament into the root canal, can enhance pulp regeneration. The migratory ability of PDLCs was significantly increased by the treatment with CE, DE and fibronectin together with 10% FBS compared to 10% FBS only (Figures 5Ca,b). There was a lower migration in the PDLCs treated with fibronectin compared with the CE and DE treatments, suggesting that other factors released in the CE and DE enhanced the cell migration.

**FIGURE 6:**
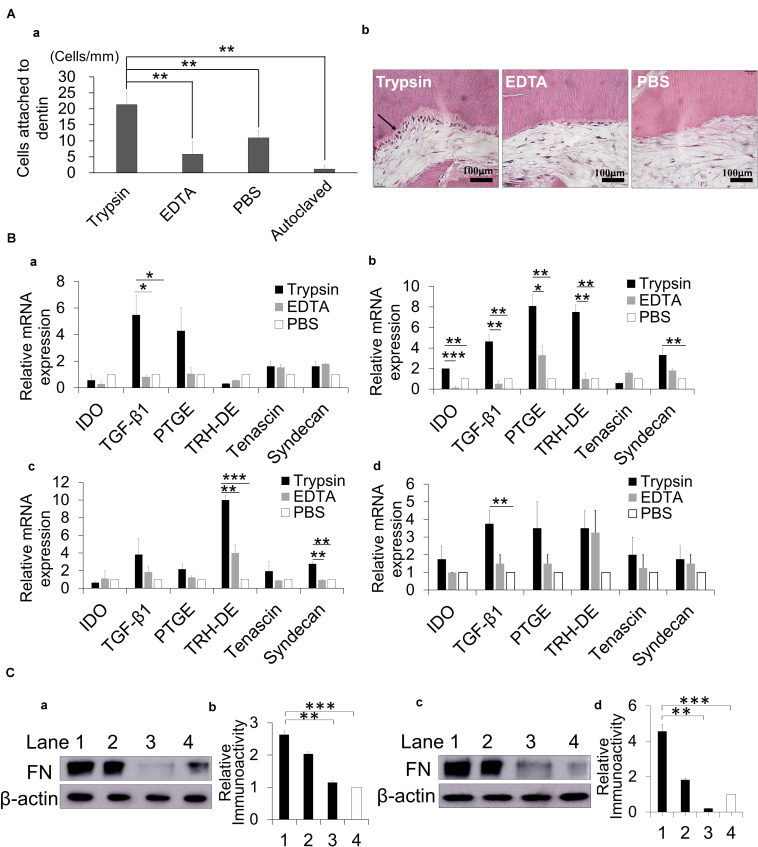
The effect of trypsin treatment of dentin compared with EDTA on cell attachment, immunomodulatory and pulp marker expressions and fibronectin (FN) release. **(A)** The number of cells attached to the dentin slice **(a)**. Cell attachment and odontoblast-like cell differentiation (arrow) on dentin treated with 0.05% trypsin at 4 weeks **(b)**. **(B)** Effect of dentin extract treated with 0.05% trypsin, 10% EDTA and PBS control on gene expression of immunomodulatory factors and pulp tissue markers by real-time PCR analyses in **(a)** young and **(b)** aged dental pulp stem cells (DPSCs), **(c)** young and **(d)** aged periodontal ligament cells (PDLCs). *p < 0.05, **p < 0.01, and ***p < 0.001. **(C)** FN expression by Western blot analyses in **(a,b)** young dentin extract and **(c,d)** aged dentin extract treated with 0.05% trypsin (lane 1), 0.05% chymotrypsin (lane 2), 10% EDTA (lane 3), and PBS control (lane 4) for 30 min. **(b,d)** The quantitative analyses of FN. **p < 0.01, ***p < 0.001. All data are expressed as the means ± standard deviation (*n* = 3).

Furthermore, the trypsin-treated dentin slice could induce significantly more cell attachment than the EDTA-treated dentin slice (Figure 6Aa). The cells attached to the dentinal wall treated with trypsin could differentiate into odontoblast-like cells by extending their processes into dentinal tubules *in vitro* (Figure 6Ab) and *in vivo* (Figures 1I–L). The mRNA expression levels of the immunomodulatory factors (*IDO*, *TGF*β*1*, and *PTGE*) and pulp tissue markers (*TRH-DE*, and *Syndecan*) were higher in the aged DPSCs treated with DE by trypsin compared with those treated with DE by EDTA and the PBS control (Figure 6Bb). These results suggested that the DE by trypsin has higher immunomodulatory and pulp-inductive effects on the aged DPSCs than the DE by EDTA.

In addition, Western blotting analysis showed that trypsin treatment significantly released more fibronectin from both young and aged DE than EDTA or PBS (Figures 6Ca–d), suggesting the key role of fibronectin to enhance pulp regeneration in the aged teeth.

## Discussion

### Optimal Conditions of Trypsin Pretreatment and the Efficacy and Safety Examinations

The primary goal of the present investigation was to determine the potential utility of a trypsin pretreatment prior to MDPSC transplantation with G-CSF for dentin-pulp regeneration in aged dogs. Trypsin is available for clinically use of debridement of infection. In this study, the amount of regenerated pulp tissue at 2 weeks was significantly increased by trypsin pretreatment compared to untreated controls in the aged teeth. We next determined the optimal concentration and duration of the trypsin pretreatment by the relative amount of regenerative pulp tissue, the number of odontoblasts aligning to the dentinal wall, and the ratios of vascularization and reinnervation areas to regenerated tissue areas. The brief treatment time and low concentration of trypsin was deliberately selected by our research for the safety and efficacy of the transplantation of MDPSCs with G-CSF. The volume of regenerated pulp tissue was similar for the 0.05% trypsin pretreatment for either 10 or 30 min. Supplementation with nanobubbles to 0.05% trypsin for 10 min increased the volume of regenerated pulp, suggesting the role of nanobubbles in enhancing the delivery of trypsin into the dentin. Additionally, the 0.05% trypsin pretreatment for 30 min might degenerate the tubular dentin structure to suppress cell attachment to the dentinal wall and their extension into dentinal tubules. Therefore, we selected the 0.05% trypsin pretreatment with nanobubbles for 10 min as the optimum condition. At 36 weeks after transplantation, the root canal space was completely filled with well-vascularized, innervated pulp tissue, which was considerably enclosed by newly formed dentin-like mineralized tissue. Furthermore, the safety of the trypsin pretreatment in pulp regeneration was confirmed by the findings of no abnormalities, no infiltration of inflammatory cells and no internal/external resorption of the tooth at 36 weeks. Thus, the potential utility of the trypsin pretreatment for enhanced pulp regeneration in the aged teeth was demonstrated.

### Direct Effects of Trypsin on Enhancing Pulp Regeneration in Aged Teeth

The previous work from our laboratory demonstrated that the transplanted MDPSCs produced multiple angiogenic/neurotrophic factors without being incorporated into vessels and nerves and were localized in the vicinity of proliferating cells, indicating a paracrine effect on the homing of endogenous cells from the surrounding tissue of the apical root into the root canal (Iohara et al., 2013). The transplanted MDPSCs are also not differentiated into pulp tissue cells or odontoblasts. Thus, in this experiment, PDLCs were selected as a representative of resident endogenous cells in the surrounding tissue to examine the direct effect of trypsin pretreatment. PAR2 is the cardinal PARs family member that is activated by trypsin (Miike et al., 2001). In the PDLCs from aged dog teeth, the PAR2 expression was significantly higher compared with those from young dog teeth. Thus, trypsin is possibly effective in aged resident PDLCs. The underlying mechanism by which PAR2 controls tissue regeneration was explained through 1) the inhibition of inflammation and stimulation of the repair-associated response (van den Hengel et al., 2013), 2) the inhibition of cytokine-induced apoptosis (Iablokov et al., 2014), and 3) the secretion of proangiogenic factors such as vascular endothelial growth factor (VEGF) (Rasmussen et al., 2012). The activation of PAR2 by trypsin was shown to increase the expression of the proinflammatory mediator cyclo-oxygenase-2 (COX-2), which modulates pulpal inflammation (Lundy et al., 2010). Microarray analysis or real-time PCR of the PDLCs from aged dog periapical tissues after direct treatment with trypsin showed high expression levels of genes that are possibly involved in immunomodulation/anti-inflammation, anti-apoptosis, senescence, ECM degradation, neurite outgrowth, angiogenesis, cell adhesion, and mobilization. With increasing age, there is an increase in the apoptosis of dental cells that affect the regeneration ability in aged teeth (Iezzi et al., 2019). Therefore, it is important to stimulate anti-apoptosis factors in the aged cells. Activation of PAR2 could increase MCL-1, an anti-apoptotic Bcl-2 family member (Iablokov et al., 2014) and Bcl-2 like protein 12 expression (Ma et al., 2019). In consistent, our results showed that trypsin activated aged PDLCs to release more PAR2 compared to young PDLCs and stimulated expression of the anti-apoptotic factor Bcl2 in the aged PDLCs but not in the young PDLCs. These results suggested that trypsin treatment increased the expression levels of tissue repair-related factors, anti-inflammatory factors and anti-apoptotic factors through PAR2 to promote pulp regeneration in aged teeth. On the other hand, trypsin did not have any effect on pulp regeneration in young teeth, which is consistent with the lower expression of PAR2 in the young PDLCs.

### Indirect Effects of Trypsin Pretreatment on Enhancing Pulp Regeneration in Aged Teeth

We identified by proteomic analysis many cytoskeletal components and ECM proteins, including fibronectin, in the extract from aged dog cementum treated with trypsin. Fibronectin is also detected in human cementum by proteomic analysis (Salmon et al., 2013). More fibronectin was extracted from the aged cementum compared with young cementum in dogs. The CE induced angiogenesis and neurite extension, similar to fibronectin. Fibronectin can promote neovascularization (Mongiat et al., 2016), neurite outgrowth (Tonge et al., 2012), and the migration of PDLCs (Kapila et al., 1998; Kawamura et al., 2019). Therefore, fibronectin might be the prominent factor for the enhanced angiogenesis and neurite extension in the aged teeth. However, fibronectin had less effect on the migration activity compared with CE treated with trypsin. Other migration factors, including Filamin A, Vinculin, prostacyclin synthetase, and Alpha-actinin-1, 4 (Baldassarre et al., 2009; Shao et al., 2010; Lee et al., 2019), were identified by the proteomic analysis, suggesting the mechanism for the higher migration activity of the CE than fibronectin. These factors are also reported to induce anti-apoptosis and/or proliferation (Magro et al., 2007; Lian et al., 2016). Our previous data demonstrated that PDLCs isolated from aged dog teeth had lower anti-apoptosis and proliferation abilities compared with the PDLCs from young dog teeth (Iohara et al., 2014). Thus, these findings suggested that the trypsin-treated CE containing these factors and fibronectin might rejuvenate the resident cells in the surrounding tissue of aged teeth by enhancing cell survival by anti-apoptosis, migration, and proliferation, and promoting angiogenesis and neurite extension.

EDTA treatment is demonstrated to release growth factors, especially TGF-β1, from dentin and to promote the adhesion, migration and differentiation of DPSCs into odontoblast-like cells on dentin (Galler et al., 2016). The present *in vitro* study demonstrated that the trypsin treatment could induce more DPSCs attachment to the walls of sliced dentine compared with EDTA on day 1. Our pellet culture experiment demonstrated that DPSCs attached to the dentinal wall of dentin particles treated with trypsin could differentiate into odontoblast-like cells at 4 weeks, indicating the potential odontoblastic differentiation of the attached cells on the dentinal wall treated with trypsin. The DE treated with trypsin induced higher expression levels of anti-inflammatory factors and pulp markers in the aged DPSCs compared with EDTA. Furthermore, the aged DE treated with trypsin was also demonstrated to release more fibronectin than EDTA and to enhance cell migration, angiogenesis and neurite extension. These results suggested a larger anti-inflammatory role of trypsin in the regenerated pulp tissue and demonstrated their higher contributions to enhanced cell attachment, odontoblastic differentiation and pulp regeneration induced by highly migrating resident stem cells from the surrounding tissue of the aged teeth compared with those seen after EDTA treatment. Thus, the trypsin treatment might be superior to EDTA treatment for stimulating pulp regeneration in aged teeth.

In summary, pulp regeneration was enhanced in aged dog teeth by trypsin pretreatment. The direct effects of trypsin pretreatment may be enhanced by anti-apoptosis activity and increased expression of various growth factors/cytokines via PAR2 in the aged PDLCs. The CE and DE, which include fibronectin, stimulate the migration of resident PDLCs into the root canal and enhance angiogenesis and neurite extension. After treatment with trypsin, the DE presents effects that are superior to those obtained by EDTA treatment, including anti-inflammation, pulp induction and odontoblastic differentiation effects.

## Data Availability Statement

All datasets generated for this study are included in the article/Supplementary Material.

## Ethics Statement

The animal study was reviewed and approved by the Animal Care and Use Committee of the National Center for Geriatrics and Gerontology, Research Institute and the Aichi Medical University (permission # 2016-5, 2017-25).

## Author Contributions

KI: conception and design, provision of study materials, collection and assembly of data, data analysis, and manuscript writing. MZ, YT, and HW: collection of data, data analysis and interpretation, manuscript and writing. MN: conception and design, financial support, collection and assembly of data, data analysis and interpretation, manuscript writing, and final approval of the manuscript. All authors contributed to the article and approved the submitted version.

## Conflict of Interest

MN was employed by Aeras Bio Inc., Air Water Group. The remaining authors declare that the research was conducted in the absence of any commercial or financial relationships that could be construed as a potential conflict of interest.

## References

[B1] Baldassarre M. Razinia Z. Burande C. F. Lamsoul I. Lutz P. G. Calderwood D. A. (2009). Filamins regulate cell spreading and initiation of cell migration. *PLoS One* 4:e7830. 10.1371/journal.pone.0007830 19915675 PMC2773003

[B2] Blau H. M. Cosgrove B. D. Ho A. T. (2015). The central role of muscle stem cells in regenerative failure with aging. *Nat. Med.* 21 854–862. 10.1038/nm.3918 26248268 PMC4731230

[B3] Bluff J. E. Brown N. J. Reed M. W. R. Staton C. A. (2008). Tissue factor, angiogenesis and tumour progression. *Breast Cancer Res. BCR* 10 204–204. 10.1186/bcr1871 18373885 PMC2397518

[B4] Chen J. Maniwa S. Ochi M. (2000). Influence of trypsin on the biological bonding of cartilaginous surface to bone in rabbits. *Arch. Orthop. Trauma Surg.* 120 587–591. 10.1007/s004020000139 11110142

[B5] Galler K. M. Widbiller M. Buchalla W. Eidt A. Hiller K. A. Hoffer P. C. (2016). EDTA conditioning of dentine promotes adhesion, migration and differentiation of dental pulp stem cells. *Int. Endod. J.* 49 581–590. 10.1111/iej.12492 26114662

[B6] Gibon E. Lu L. Goodman S. B. (2016). Aging, inflammation, stem cells, and bone healing. *Stem Cell Res. Ther.* 7:44 10.1186/s13287-016-0300-309 PMC4804630 27006071

[B7] Horibe H. Murakami M. Iohara K. Hayashi Y. Takeuchi N. Takei Y. (2014). Isolation of a stable subpopulation of mobilized dental pulp stem cells (MDPSCs) with high proliferation, migration, and regeneration potential is independent of age. *PLoS One* 9:e98553. 10.1371/journal.pone.0098553 24870376 PMC4037225

[B8] Huttner E. A. Machado D. C. de Oliveira R. B. Antunes A. G. Hebling E. (2009). Effects of human aging on periodontal tissues. *Spec. Care Dent.* 29 149–155. 10.1111/j.1754-4505.2009.00082.x 19573041

[B9] Iablokov V. Hirota C. L. Peplowski M. A. Ramachandran R. Mihara K. Hollenberg M. D. (2014). Proteinase-activated receptor 2 (PAR2) decreases apoptosis in colonic epithelial cells. *J. Biol. Chem.* 289 34366–34377. 10.1074/jbc.M114.610485 25331954 PMC4256365

[B10] Iezzi I. Pagella P. Mattioli-Belmonte M. Mitsiadis T. A. (2019). The effects of ageing on dental pulp stem cells, the tooth longevity elixir. *Eur. Cell Mater.* 37 175–185. 10.22203/eCM.v037a11 30805914

[B11] Iohara K. Murakami M. Nakata K. Nakashima M. (2014). Age-dependent decline in dental pulp regeneration after pulpectomy in dogs. *Exp. Gerontol.* 52 39–45. 10.1016/j.exger.2014.01.020 24468330

[B12] Iohara K. Murakami M. Takeuchi N. Osako Y. Ito M. Ishizaka R. (2013). A novel combinatorial therapy with pulp stem cells and granulocyte colony-stimulating factor for total pulp regeneration. *Stem Cells Transl. Med.* 2:818. 10.5966/sctm.2012-0132erratum 28945010 PMC3785266

[B13] Iohara K. Nakashima M. (2019). Enhanced delivery of EDTA by nanobubbles into dentin for efficient demineralization to enlarge the constricted root canal. *Jpn. J. Conserv. Dent.* 62 152–158. 10.11471/shikahozon.62.152

[B14] Ishizaka R. Iohara K. Murakami M. Fukuta O. Nakashima M. (2012). Regeneration of dental pulp following pulpectomy by fractionated stem/progenitor cells from bone marrow and adipose tissue. *Biomaterials* 33 2109–2118. 10.1016/j.biomaterials.2011.11.056 22177838

[B15] Jang A. T. Lin J. D. Choi R. M. Choi E. M. Seto M. L. Ryder M. I. (2014). Adaptive properties of human cementum and cementum dentin junction with age. *J. Mech. Behav. Biomed. Mater.* 39 184–196. 10.1016/j.jmbbm.2014.07.015 25133753 PMC4265544

[B16] Jurk D. Wilson C. Passos J. F. Oakley F. Correia-Melo C. Greaves L. (2014). Chronic inflammation induces telomere dysfunction and accelerates ageing in mice. *Nat. Commun.* 5:4172. 10.1038/ncomms5172 24960204 PMC4090717

[B17] Kapila Y. L. Lancero H. Johnson P. W. (1998). The response of periodontal ligament cells to fibronectin. *J. Periodontol.* 69 1008–1019. 10.1902/jop.1998.69.9.1008 9776029

[B18] Kawamura M. Yamamoto T. Yamashiro K. Kochi S. Yoshihara-Hirata C. Ideguchi H. (2019). Induction of migration of periodontal ligament cells by selective regulation of integrin subunits. *J. Cell Mol. Med.* 23 1211–1223. 10.1111/jcmm.14023 30511442 PMC6349235

[B19] Krieger E. Hornikel S. Wehrbein H. (2013). Age-related changes of fibroblast density in the human periodontal ligament. *Head Face Med.* 9:22. 10.1186/1746-160X-9-22 23965233 PMC3844409

[B20] Lee H. T. Sharek L. O’Brien E. T. Urbina F. L. Gupton S. L. Superfine R. (2019). Vinculin and metavinculin exhibit distinct effects on focal adhesion properties, cell migration, and mechanotransduction. *PLoS One* 14:e0221962. 10.1371/journal.pone.0221962 31483833 PMC6726196

[B21] Lian G. Dettenhofer M. Lu J. Downing M. Chenn A. Wong T. (2016). Filamin A- and formin 2-dependent endocytosis regulates proliferation via the canonical Wnt pathway. *Development* 143:4509. 10.1242/dev.139295 27789627 PMC5201043

[B22] Lundy F. T. About I. Curtis T. M. McGahon M. K. Linden G. J. Irwin C. R. (2010). PAR-2 regulates dental pulp inflammation associated with caries. *J. Dent. Res.* 89 684–688. 10.1177/0022034510365652 20505052

[B23] Ma G. Wang C. Lv B. Jiang Y. Wang L. (2019). Proteinase-activated receptor-2 enhances Bcl2-like protein-12 expression in lung cancer cells to suppress p53 expression. *Arch. Med. Sci.* 15 1147–1153. 10.5114/aoms.2019.86980 31572459 PMC6764318

[B24] Magro A. M. Magro A. D. Cunningham C. Miller M. R. (2007). Down-regulation of vinculin upon MK886-induced apoptosis in LN18 glioblastoma cells. *Neoplasma* 54 517–526. 17949236 PMC4320946

[B25] Miike S. McWilliam A. S. Kita H. (2001). Trypsin induces activation and inflammatory mediator release from human eosinophils through protease-activated receptor-2. *J. Immunol.* 167 6615–6622. 10.4049/jimmunol.167.11.6615 11714832

[B26] Mongiat M. Andreuzzi E. Tarticchio G. Paulitti A. (2016). Extracellular matrix, a hard player in angiogenesis. *Int. J. Mol. Sci.* 17:1822. 10.3390/ijms17111822 27809279 PMC5133823

[B27] Murakami M. Horibe H. Iohara K. Hayashi Y. Osako Y. Takei Y. (2013). The use of granulocyte-colony stimulating factor induced mobilization for isolation of dental pulp stem cells with high regenerative potential. *Biomaterials* 34 9036–9047. 10.1016/j.biomaterials.2013.08.011 23988014

[B28] Murakami M. Imabayashi K. Watanabe A. Takeuchi N. Ishizaka R. Iohara K. (2012). Identification of novel function of vimentin for quality standard for regenerated pulp tissue. *J. Endod.* 38 920–926. 10.1016/j.joen.2012.01.010 22703654

[B29] Nakashima M. Iohara K. (2017). Recent progress in translation from bench to a pilot clinical study on total pulp regeneration. *J. Endod.* 43(9, Suppl.), S82–S86. 10.1016/j.joen.2017.06.014 28778509

[B30] Nakashima M. Iohara K. Murakami M. Nakamura H. Sato Y. Ariji Y. (2017). Pulp regeneration by transplantation of dental pulp stem cells in pulpitis: a pilot clinical study. *Stem Cell Res. Ther.* 8:61 10.1186/s13287-017-0506-505 PMC5345141 28279187

[B31] Qin C. Brunn J. C. Cook R. G. Orkiszewski R. S. Malone J. P. Veis A. (2003). Evidence for the proteolytic processing of dentin matrix protein 1. Identification and characterization of processed fragments and cleavage sites. *J. Biol. Chem.* 278 34700–34708. 10.1074/jbc.M305315200 12813042

[B32] Rajendran S. (2018). *Advanced Textiles for Wound Care.* Sawston: Woodhead Publishing.

[B33] Ramachandran R. Altier C. Oikonomopoulou K. Hollenberg M. D. (2016). Proteinases, their extracellular targets, and inflammatory signaling. *Pharmacol. Rev.* 68 1110–1142. 10.1124/pr.115.010991 27677721

[B34] Ramelli G. Fuertes S. Narayan S. Busso N. Acha-Orbea H. So A. (2010). Protease-activated receptor 2 signalling promotes dendritic cell antigen transport and T-cell activation in vivo. *Immunology* 129 20–27. 10.1111/j.1365-2567.2009.03144.x 19845798 PMC2807483

[B35] Rasmussen J. G. Riis S. E. Frøbert O. Yang S. Kastrup J. Zachar V. (2012). Activation of protease-activated receptor 2 induces VEGF independently of HIF-1. *PLoS One* 7:e46087. 10.1371/journal.pone.0046087 23049945 PMC3457954

[B36] Rovai E. S. Holzhausen M. (2017). The role of proteinase-activated receptors 1 and 2 in the regulation of periodontal tissue metabolism and disease. *J. Immunol. Res.* 2017:5193572. 10.1155/2017/5193572 28503577 PMC5414592

[B37] Salmon C. R. Tomazela D. M. Ruiz K. G. S. Foster B. L. Paes Leme A. F. Sallum E. A. (2013). Proteomic analysis of human dental cementum and alveolar bone. *J. Proteomics* 91 544–555. 10.1016/j.jprot.2013.08.016 24007660 PMC3873800

[B38] Schmalz G. Widbiller M. Galler K. M. (2017). Signaling molecules and pulp regeneration. *J. Endod.* 43 S7–S11. 10.1016/j.joen.2017.06.003 28844306

[B39] Shah D. Mital K. (2018). The role of trypsin:chymotrypsin in tissue repair. *Adv. Ther.* 35 31–42. 10.1007/s12325-017-0648-y 29209994 PMC5778189

[B40] Shao H. Wang J. H. C. Pollak M. R. Wells A. (2010). α-Actinin-4 is essential for maintaining the spreading, motility and contractility of fibroblasts. *PLoS One* 5:e13921. 10.1371/journal.pone.0013921 21085685 PMC2978680

[B41] Silva T. A. Rosa A. L. Lara V. S. (2004). Dentin matrix proteins and soluble factors: intrinsic regulatory signals for healing and resorption of dental and periodontal tissues? *Oral Dis.* 10 63–74. 10.1111/j.1601-0825.2004.00992.x 14996275

[B42] Smith A. J. (2003). Vitality of the dentin-pulp complex in health and disease: growth factors as key mediators. *J. Dent. Educ.* 67 678–689. 10.1002/j.0022-0337.2003.67.6.tb03668.x 12856968

[B43] Smith A. J. Scheven B. A. Takahashi Y. Ferracane J. L. Shelton R. M. Cooper P. R. (2012). Dentine as a bioactive extracellular matrix. *Arch. Oral. Biol.* 57 109–121. 10.1016/j.archoralbio.2011.07.008 21855856

[B44] Tonge D. A. de Burgh H. T. Docherty R. Humphries M. J. Craig S. E. Pizzey J. (2012). Fibronectin supports neurite outgrowth and axonal regeneration of adult brain neurons in vitro. *Brain Res.* 1453 8–16. 10.1016/j.brainres.2012.03.024 22483961 PMC3989037

[B45] van den Hengel L. G. Hellingman A. A. Nossent A. Y. van Oeveren-Rietdijk A. M. de Vries M. R. Spek C. A. (2013). Protease-activated receptor (PAR)2, but not PAR1, is involved in collateral formation and anti-inflammatory monocyte polarization in a mouse hind limb ischemia model. *PLoS One* 8:e61923. 10.1371/journal.pone.0061923 23637930 PMC3630144

[B46] Widbiller M. Eidt A. Wolflick M. Lindner S. R. Schweikl H. Hiller K. A. (2018). Interactive effects of LPS and dentine matrix proteins on human dental pulp stem cells. *Int. Endod. J.* 51 877–888. 10.1111/iej.12897 29377169

[B47] Zayed M. Iohara K. Watanabe H. Nakashima M. (2020). CCR3 antagonist protects against induced cellular senescence and promotes rejuvenation in periodontal ligament cells for stimulating pulp regeneration in the aged dog. *Sci. Rep.* 10:8631 10.1038/s41598-020-65301-65309 PMC7248074 32451381

[B48] Zhao P. Lieu T. Barlow N. Metcalf M. Veldhuis N. A. Jensen D. D. (2014). Cathepsin S causes inflammatory pain via biased agonism of PAR2 and TRPV4. *J. Biol. Chem.* 289 27215–27234. 10.1074/jbc.M114.599712 25118282 PMC4175355

