# Supplementary material for: Treatment of Pulpectomized Teeth With Trypsin Prior to Transplantation of Mobilized Dental Pulp Stem Cells Enhances Pulp Regeneration in Aged Dogs
Source: Front Bioeng Biotechnol. 2020 Aug 14;8:983. doi: 10.3389/fbioe.2020.00983 (PMC7456913; doi:10.3389/fbioe.2020.00983)
Supplement: Supplementary file 1 [file Data_Sheet_1.pdf]

Supplementary Materials

Microarray analysis

Microarray analysis was performed as described previously (Ishizaka et al., 2012) with slight modification. Briefly, biotinylated cRNA were prepared from 250 ng of total RNA of directly treated aged PDLCs according to the standard Affymetrix protocol (Affymetrix Japan K.K., Japan). Subsequently fragmentation, 10 μg of cRNA were hybridized for 16 hours at 45°C on GeneChip Canine Genome 2.0 Array (Affymetrix) containing 43,000 annotated sequences. GeneChips were washed and stained in the Affymetrix Fluidics Station 450 and were scanned using the Affymetrix GCS3000 scanner. The data were analyzed with Microarray Suite version 5.0 (MAS 5.0) using Affymetrix default analysis settings and global scaling as normalization method. The trimmed mean target intensity of each array was set to 500. Chips were ordered into hierarchical clusters using the Pearson centered algorithm as the distance measure, and the average algorithm as the linkage method.

Proteomic analysis

For MS analysis, after transfer the protein spots to microtubes, the gel pieces were reduced with the buffer of 50 mmol/L NH4HCO3 containing with 10 mmol/L EDTA, and 10 mmol/L Dithiothreitol (DTT) for 1 hours at 65°C and alkylated with 50 mmol/L NH4HCO3 containing with 10 mmol/L EDTA, and 40 mmol/L iodoacetamide for 30 min at room temperature in the dark place. Those gel pieces were washed in 50 mmol/L NH4HCO3 buffer for 30 min at room temperature twice and dehydrated with acetonitrile for 10 min at room temperature. Each sample was digested with trypsin (4 mg/ml, Trypsin Gold; Promega, San Luis Obispo, CA) in 40 mmol/L NH4HCO3/10% acetonitrile at 37°C overnights. The gel pieces were further extracted with 5% formic acid/10% acetonitrile at room temperature for 10 min twice. These extracted liquids were dried under vacuum and rehydrated in 0.1% trifluoroacetic acid. The extracted peptides were purified and concentrated by ZipTip C18 pipette tips (Millipore, Billerica, MA) according to the manufacturer's instructions. The purified peptides were then separated via nano LC (Paradigm MS4; Michrom BioResources, Inc, Auburn, CA) using a Magic C18 column (0.2 × 50 mm; Michrom BioResources, Inc). The LC eluent was analyzed by LCQ Advantage MAX mass spectrometer (Thermo Fisher Scientific, Waltham, MA) equipped with an ion-spray source. All of the MS/MS spectra were searched by the SEQUEST algorithm from BioWorks software (Thermo Fisher Scientific).

Supplementary Figure 1.

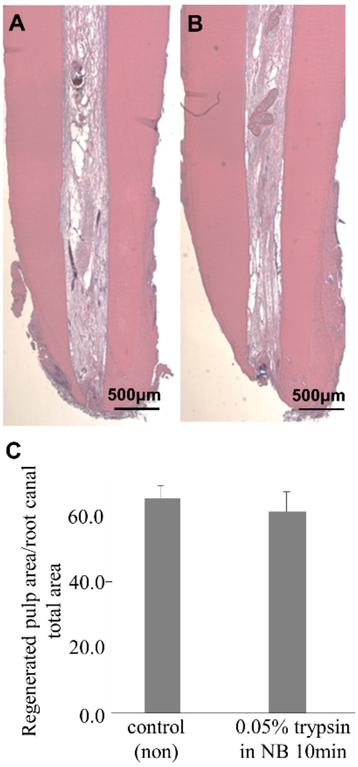

Supplemental Figure 1 Trypsin pretreatment in the pulpectomized teeth prior to cell transplantation in young dogs. **(A)** Control **(B)** 0.05% trypsin for 10min. **(C)** No significant effect of trypsin on promoting pulp regeneration in young dogs.

Supplementary Table 1. Highly expressed genes in the aged dog periodontal ligament cells treated with trypsin compared to non-treated cells by microarray analysis.

	Gene name	Relative expression Trypsin treated/non treated
ECM	THBS1	33
	EFEMP1	27
	VWA3B	22
	LRRC36	14
Cytoskeletal components	KLHL1	22
	MYH3	14
	KIF21B	15
Cytoplasmic proteins	HSP90	14
Enzyme	AMPD2	20
	MMP26	16
Growth factors/cytokines	NCF1	19
	IL18	17
Cell membrane proteins	CD69	33
	TOM1L1	32
	CNR2/CB2R	22
	RYR1	15
	IL2RB	13
	PTH2R	13
Mitochondrial protein	MARC1	16

THBS1, Thrombospondin 1; EFEMP1, EGF-containing fibulin-like extracellular matrix protein 1; VWA3B, Von Willebrand Factor A domain containing 3B; LRRC36, Leucine rich repeat containing 36; KLHL1, Kelch like family member 1, MYH3; myosin heavy chain 3, KIF21B; kinesin family member 21B, HSP90; Heat shock protein 90, AMPD2, adenosine monophosphate deaminase 2, MMP26; matrix metalloproteinase 26, NCF1; Neutrophil cytosolic factor 1, IL18; Interleukin-18, CD69; Cluster of differentiation 69, TOM1L1; target of myb1 like 1 membrane trafficking protein, CNR2/CB2R; Cannabinoid receptor 2, RYR1; Ryanodine receptor 1, IL2RB; Interleukin 2 receptor subunit beta, PTH2R; Parathyroid hormone 2 receptor, MARC1; Mitochondrial amidoxime reducing component 1.

Supplementary Table 2. Highly expressed proteins in the aged dog cementum extract treated with 0.05% trypsin compared to young dog cementum extract by proteomic analysis.

	Protein name	Relative Expression Aged cementum extract/ Young cementum extract
ECM	Fibronectin	18
	Collagen alpha-1 (XII)	17
	Collagen alpha-3 (VI)	13
Cytoskeletal component	Filamin A	30
	Cytoplasmic dynein 1 heavy chain	26
	Plectin	26
	Talin 1	18
	Alpha-actinin-4	11
	Microtubule-associated protein 1B	10
	Vinculin	9
	Myosin-9	9
	Dihydropyrimidinase-related protein 2	9
	Alpha-actinin-1	8
	Myosin-10	6
	Septin-2	4
Signaling molecules	Clathrin heavy chain 1	15
Regulatory protein	Prostacyclin synthetase	4
	Aspartyl aminopeptidase	4
